# Identifying missing pieces in color vision defects: a genome-wide association study in Silk Road populations

**DOI:** 10.3389/fgene.2023.1161696

**Published:** 2023-06-09

**Authors:** Giuseppe Giovanni Nardone, Beatrice Spedicati, Maria Pina Concas, Aurora Santin, Anna Morgan, Lorenzo Mazzetto, Maurizio Battaglia-Parodi, Giorgia Girotto

**Affiliations:** ^1^ Department of Medicine, Surgery and Health Sciences, University of Trieste, Trieste, Italy; ^2^ Institute for Maternal and Child Health - IRCCS “Burlo Garofolo”, Trieste, Italy; ^3^ Ophthalmology Department, Vita-Salute San Raffaele University, Milano, Italy

**Keywords:** color vision defects, Deutan, Protan, Tritan, genome wide association study, genetic isolates, Silk Road, pathway analysis

## Abstract

**Introduction:** Color vision defects (CVDs) are conditions characterized by the alteration of normal trichromatic vision. CVDs can arise as the result of alterations in three genes (*OPN1LW*, *OPN1MW*, *OPN1SW*) or as a combination of genetic predisposition and environmental factors. To date, apart from Mendelian CVDs forms, nothing is known about multifactorial CVDs forms.

**Materials and Methods:** Five hundred and twenty individuals from Silk Road isolated communities were genotyped and phenotypically characterized for CVDs using the Farnsworth D-15 color test. The CVDs traits Deutan-Protan (DP) and Tritan (TR) were analysed. Genome Wide Association Study for both traits was performed, and results were corrected with a False Discovery Rate linkage-based approach (FDR-p). Gene expression of final candidates was investigated using a published human eye dataset, and pathway analysis was performed.

**Results:** Concerning DP, three genes: *PIWIL4* (FDR-p: 9.01*10^–9^), *MBD2* (FDR-p: 4.97*10^–8^) and *NTN1* (FDR-p: 4.98*10^–8^), stood out as promising candidates. *PIWIL4* is involved in the preservation of Retinal Pigmented Epithelium (RPE) homeostasis while *MBD2* and *NTN1* are both involved in visual signal transmission. With regards to TR, four genes: *VPS54* (FDR-p: 4.09*10^–9^), *IQGAP* (FDR-p: 6,52*10^–10^), *NMB* (FDR-p: 8.34*10^–11^), and *MC5R* (FDR-p: 2.10*10^–8^), were considered promising candidates. *VPS54* is reported to be associated with Retinitis pigmentosa; *IQGAP1* is reported to regulate choroidal vascularization in Age-Related Macular Degeneration; *NMB* is involved in RPE homeostasis regulation; *MC5R* is reported to regulate lacrimal gland function.

**Discussion:** Overall, these results provide novel insights regarding a complex phenotype (i.e., CVDs) in an underrepresented population such as Silk Road isolated communities.

## 1 Introduction

Color vision defines an organism’s ability to differentiate objects by wavelengths of light that they reflect, emit, or transmit. Many differences exist between animal and human color vision. Human vision is trichromatic, while some animals, such as frogs, fish, marsupials and some types of rodents, can perceive ultraviolet. Birds are believed to possess the most sophisticated visual system among vertebrates, probably perceiving hues of colors inaccessible to humans. In humans, color vision involves several retina and brain mechanisms. In particular, light-absorbing molecules defined as visual pigments, consisting of integral membrane proteins in photoreceptor cells, mediate human vision. The energy carried by photons activates the pigments and eventually starts the photo transduction signal cascade, thus converting the light into an electric signal. Visual pigments reside in two types of photoreceptors, the cone-shaped and rod-shaped cells, and are classified according to the wavelength of maximum light absorption (λ_max_). Cone photoreceptors contain the three primary visual pigments, the short-wavelength sensitive (S; λ_max_ ∼420 nm), the medium-wavelength sensitive (M; λ_max_ ∼530 nm), and the long-wavelength sensitive (L; λ_max_ ∼560 nm), while rod-shaped cells contain a fourth pigment, called rhodopsin, which mediates vision in dim light conditions and absorbs maximally at λ_max_ ∼495 nm (Nathans, 1999). Humans, along with primates, possess three genes encoding for the three primary visual pigments: *OPN1SW*, located on chromosome 7, and *OPN1MW* and *OPN1LW*, both located on chromosome X. Trichromacy arises with the duplication of the *OPN1LW* gene resulting in the formation of the *OPN1MW* gene (Dulai et al., 1999), which confers the ability to discriminate red fruits on a green background. Interestingly, the acquisition of trichromatic vision concurred with the deterioration of the olfactory system in primates (Gilad et al., 2004).

Color Vision Defects (CVDs) are conditions caused by the lack of visual pigments or by their abnormal function. CVDs are divided into congenital and acquired forms and classified based on the affected cone: Deutan affects M-cones causing a defective perception of the red-yellow-green spectrum; Protan affects L-cones causing poor discrimination of the red-green spectrum; and Tritan affects S-cones leading to an alteration in the perception of blue, yellow and orange hues (Hasrod and Rubin, 2016). Congenital CVDs (Deuteranopia, Protanopia, and Tritanopia) are the most characterised forms. They are caused by alterations in: genes coding visual pigments; genes controlling their expression; genes coding components of the phototransduction cascade; genes coding for the α- or β-subunits of the cone cyclic guanosine monophosphate–gated cation channels. Conversely, acquired CVDs arise as a result of ocular, neurologic, or systemic diseases. They can be classified based on the primary disease and the type of color vision alteration encountered (Simunovic, 2016). Moreover, color discrimination ability starts gradually declining from the age of 30. In addition, CVDs can arise during the ageing process, may be influenced by pupil size, crystalline lens coloration, and macular pigment, suggesting the existence of multifactorial forms of CVDs (Ichikawa et al., 2021).

In recent years, Genome-Wide Association Studies (GWAS) proved to be a valid tool in the discovery of novel genes associated with complex diseases and multifactorial traits (Struck et al., 2018). Moreover, it has been proven that GWAS discovery power can be increased by investigating genetic isolates - small communities with reduced genetic variation and environmental homogeneity - offering the possibility to highlight variants underlying complex traits (Girotto et al., 2011). Here, we present the first GWAS analysis investigating multifactorial CVDs in genetic isolates from the Silk Road countries. For centuries, the Silk Road has been a crucial trading route directly linking Europe with Asia. Due to its geographical location and past socio-economic relevance, today, the Silk Road represents a unique collection of populations with admixed ancestries (Mezzavilla et al., 2014), languages and traditions, thus offering the possibility to study rare and unique patterns of genetic variation. Therefore, this study aims to identify novel variants and genes potentially involved in the determination of multifactorial CVDs. To reach this goal, we performed 1) GWAS analyses, 2) evaluation of the expression of candidate genes in a published database, and 3) up-to-date *in silico* pathway construction to evaluate interactions between candidate genes and known genes involved in eye physiology and pathology.

## 2 Materials and methods

### 2.1 Cohort characteristics

Eight hundred ninety-three subjects from 20 isolated communities spread across nine Central Asia and Caucasus countries (Afghanistan, Armenia, Azerbaijan, Crimea, Georgia, Kazakistan, Kirghizistan, Tajikistan, Uzbekistan) were recruited during the “Marco Polo” scientific expedition in 2010 (www.marcopolo2010.it). Biological samples, along with information about age, sex, lifestyle, eating habits, profession, and smoking and alcohol consumption, were collected. Moreover, phenotypic information about sensorial abilities and performance, such as hearing thresholds, olfactory performance, and food preferences were collected using several tests and questionnaires. Written informed consent was obtained from all enrolled subjects. The study was conducted in accordance with the Helsinki Declaration, and the research protocol was approved by the ethical committee of IRCCS “Burlo Garofolo” of Trieste, Italy.

### 2.2 Color vision evaluation

Participants were tested for color discrimination ability using the Farnsworth D-15 saturated color test, administered to all subjects in natural daylight lighting conditions (Oli and Joshi, 2019). Briefly, 15 enumerated colored disks and a reference disk are used in the test. First, subjects select the disk that most closely matches the reference. Then, they select the next color disk matching the previous one and continue until the last disk has been positioned. Scoring is defined by reading the number on the rear of the disk and diagramming the subject’s results on a template sheet. Lines crossing the center of the diagram define the type and severity of the CVD (Supplementary Figure S1). To obtain a more precise classification, the tests’ results interpretation was carried out by a certified ophthalmologist. Individuals were finally classified in Deutan-Protan (DP, 31 cases) and Tritan (TR, 12 cases). All the individuals without color vision alteration were classified as controls.

### 2.3 Genotyping and imputation

Saliva samples were collected from all enrolled subjects using the Oragene DNA collection kit, and DNA extraction was performed (DNA Genotek, Ontario, Canada). Genotyping was carried out using the Omni Express 700k Illumina Chip, including only samples and sites with call rate ≥0.99 (43,655 variants excluded), Hardy-Weinberg Equilibrium *p*-value ≥1*10^–6^ (3,153 variants excluded), and minor allele frequency (MAF) ≥ 0.01 (18 variants excluded). Imputation was performed using MINIMAC v4 (Howie et al., 2012) to Haplotype Reference Consortium imputation panel version r1.1 (McCarthy et al., 2016). All data were aligned to the human reference genome build 37 (GRCh37). After imputation, SNPs with Info Score <0.4 (13626943 variants excluded) and MAF <0.01 (40,203 variants excluded) were discarded from statistical analyses. A total of 7783479 SNPs were analysed.

### 2.4 Genome-wide association studies (GWAS)

GWAS analyses for DP and TR traits were conducted using GEMMA v0.98 software (Zhou and Stephens, 2012). Linear mixed model regressions assuming an additive genetic model were performed. The genomic kinship matrix was used as random effects to take into account relatedness. Covariates included in the analyses were: age, sex, educational level and the first ten principal components (PCs). PCs were calculated from 203,099 genotyped variants using Plink v1.9 (Purcell et al., 2007). Results were then adjusted using a False Discovery Rate (FDR) linkage-based approach adapted from (Chen et al., 2021). Briefly, linkage disequilibrium-based clumping of regression results was performed using Plink v1.9, setting an *r* ^2^ threshold of 0.3 and a variant distance of 0.5 Mb. Then, the most significant SNPs of each clump were collected in a new dataset, and FDR calculation was carried out using R (https://www.r-project.org/index.html). For each GWAS, SNPs with an FDR-adjusted *p*-value (FDR-p) ≤ 1 × 10^−5^ were annotated with the Variant Effect Predictor tool (VEP) (McLaren et al., 2016) to obtain information on the distance from the closest genes and their functional characteristics (i.e., whether they were contained in an intronic, exonic, or intergenic region). For each SNP, the nearest coding gene in a range of 250 kb was annotated. Long non-coding RNAs (LINC) genes, genes with unknown functions identified with LOC and FAM symbols, and pseudo genes were excluded. Finally, potential candidate genes were selected based on the following criteria:

• FDR-p ≤ 5*10^–8^;
• Number of SNPs in linkage with the top SNP ≥ the median of SNPs in linkage for each clump;
• Expression levels in the inquired database;
• Involvement in physiological and/or pathological processes in the eye.

Manhattan and QQplots were generated using R, while heatmap was generated using the ComplexHeatmap R package (Gu et al., 2016).

### 2.5 Gene expression in eye tissues

The final candidate genes’ expression was investigated using a published human eye transcriptome dataset - the “Eye in a Disk” (EiaD) (https://eyeintegration.nei.nih.gov). Briefly, the dataset integrates expression data from GTEx (GTEx Consortium, 2013) and public repositories through the use of a Snakemake-based pipeline to normalise expression values indicated as log_2_ [Transcript Per Million (TPM) +1)] and perform quality control. Expression data was extracted from healthy human adult and fetal eye tissues and eye stem cells. For this study, gene expression of candidate genes in the following tissues was investigated: Corneal Endothelium (CE); Corneal Fetal Endothelium (CFE); Corneal Endothelial Stem Cells (CSE); Cornea (Cn); Lens Stem Cell Line (LSC); Fetal Eye Retina (RFE); Retinal Fetal Tissue (RFT); Retinal Ganglion Stem Cells (RGS); Retinal Pigment Epithelium (RPE); Fetal RPE (RPFT); Retina (Rt).

### 2.6 Pathway analysis

Finally, the involvement of the candidate genes in eye physiology and pathology was investigated using Qiagen’s Ingenuity Pathway Analysis (IPA) from Ingenuity Systems (Redwood City, California, United States; http://www.ingenuity.com). Briefly, a list of promising candidate genes resulting from significant FDR-corrected associations for both traits was uploaded to verify their interaction with a set of genes involved in eye pathologies and physiology using Path Explorer. Finally, interaction maps were generated using IPA’s Path Designer. The gene list was obtained by combining manual bibliographic research and IPA databases, including genes involved in cone photoreceptor disorders and cone cell development.

## 3 Results

### 3.1 GWAS results

Individuals with missing data regarding age, sex and color vision, or missing genetic data were excluded from the analyses. In the GWAS for DP, 514 individuals were included, specifically 31 with DP and 483 without color vision alteration. In the GWAS for TR, 520 subjects were considered, precisely 12 with TR and 508 without color vision alteration. The number, gender, age, and educational level of analyzed participants are reported in Table 1.

**TABLE 1:** Characteristics of the subjects involved in the study, divided by color vision defect.

	All	DP affected	Not affected	All	TR affected	Not affected
N (F)	514 (298)	31 (13)	483 (285)	520 (313)	12 (9)	508 (304)
Age						
Mean	36.4 (36.1)	43.7 (45)	36 (35.7)	36.3 (36.3)	40.1 (37.5)	36.2 (36.2)
SD	15.3 (15.1)	16.8 (21.7)	15 (14.5)	15.1 (14.7)	15.1 (14.7)	15.1 (14.7)
Educational Level - N (F)						
Elementary School	23 (18)	6 (6)	17 (12)	20 (14)	2 (2)	18 (12)
Middle School	59 (36)	4 (0)	55 (36)	58 (38)	1 (1)	57 (37)
High School	251 (146)	13 (5)	238 (141)	263 (159)	8 (5)	255 (154)
University	181 (98)	8 (2)	173 (96)	179 (102)	1 (1)	178 (101)

The number, age, and educational level of analyzed participants is reported; the number, age, and educational level of females is reported in brackets. Age is expressed through mean and standard deviation. Educational level is classified in elementary school, middle school, high school, and university. N: number; F: females; SD: standard deviation; DP: Deutan-Protan; TR: tritan.

Manhattan and QQ plots of GWAS on DP and TR are shown in Supplementary Figure S2. A total of 1,263 associations between SNPs and DP phenotype with *p* < 1*10^–5^ were detected. Of those, 103 displayed genome-wide significant *p*-values (*p* < 5*10^–8^). Regarding TR, 2,968 associations with *p* < 1*10^–5^ were found; 273 with genome-wide significant *p*-values. After the FDR correction, 489 variants with FDR-corrected *p*-values (FDR-p) < 1*10^–5^ were retained for DP. Of those, nine showed genome-wide significant FDR-p (Supplementary Table S1). Eight hundred eighty-three variants with FDR-p < 1*10^–5^ were retained for the TR trait, 45 with genome-wide significant FDR-p (Supplementary Table S1). All variants showing genome-wide significant FDR-p for both DP and TR were not in linkage-disequilibrium. VEP annotation of the association results for both traits is available in Supplementary Tables S2, S3. Following the selection criteria described in the Materials and Methods section, our analyses allowed the identification of 18 promising candidates, as displayed in Table 2.

**Table 2:** Selected genes associated with the DP (Deutan-Protan) and TR (Tritan) phenotypes.

Trait	Genes	Top SNP (rs)	HGVS nomenclature	Freq	Beta	StdErr	*p*-value	FDR-p	N° of surrounding SNPs (*p* < 1 × 10^−4^)
DP	*PIWIL4*	rs118136100	NC_000011.9:g.94309876G>A	0.983	−0.412	0.061	1.30 × 10^−10^	9.01 × 10^−9^	2
DP	*NTN1*	rs117797822	NC_000017.10:g.9111250T>C	0.970	−0.304	0.048	1.08 × 10^−9^	4.98 × 10^−8^	3
DP	*MBD2*	rs17292725	NC_000018.9:g.51880889G>A	0.959	−0.294	0.046	8.06 × 10^−10^	4.97 × 10^−8^	3
TR	*NMB*	rs145079583	NC_000015.9:g.85195862A>G	0.984	−0.344	0.043	9.45 × 10^−14^	8.34 × 10^−11^	18
TR	*ATF7IP2*	rs146729070	NC_000016.9:g.10394350C>T	0.981	−0.318	0.043	4.19 × 10^−12^	6.52 × 10^−10^	2
TR	*IQGAP1*	rs117555778	NC_000015.9:g.91019452C>T	0.975	−0.231	0.031	4.14 × 10^−12^	6.52 × 10^−10^	6
TR	*NPTN*	rs192430987	NC_000015.9:g.73933216A>C	0.974	−0.243	0.033	4.43 × 10^−12^	6.52 × 10^−10^	4
TR	*GALNT1*	rs55833596	NC_000018.9:g.33141906C>T	0.983	−0.232	0.034	6.35 × 10^−11^	4.09 × 10^−9^	2
TR	*VPS54*	rs140150162	NC_000002.11:g.64279003A>C	0.983	−0.237	0.034	6.48 × 10^−11^	4.09 × 10^−9^	340
TR	*BIN3*	rs73212812	NC_000008.10:g.22532279G>A	0.980	−0.24	0.038	1.14 × 10^−9^	2.91 × 10^−8^	4
TR	*HTR1B*	rs78752155	NC_000006.11:g.77972985C>T	0.978	−0.227	0.036	1.21 × 10^−9^	2.91 × 10^−8^	2
TR	*TCIRG1*	rs76912991	NC_000011.9:g.67823868A>G	0.970	−0.174	0.027	6.52 × 10^−10^	2.13 × 10^−8^	3
TR	*MC5R*	rs77046774	NC_000018.9:g.13825162A>G	0.944	−0.132	0.020	6.17 × 10^−10^	2.10 × 10^−8^	44
TR	*ATP2B1*	rs117316296	NC_000012.11:g.90338181A>G	0.986	−0.274	0.038	1.70 × 10^−11^	1.75 × 10^−9^	2
TR	*BBX*	rs116495417	NC_000003.11:g.107567716C>T	0.971	−0.209	0.032	4.01 × 10^−10^	1.54 × 10^−8^	2
TR	*MDGA2*	rs72680270	NC_000014.8:g.47740920G>T	0.976	−0.219	0.033	3.54 × 10^−10^	1.42 × 10^−8^	7
TR	*HCN4*	rs191157379	NC_000015.9:g.73500591G>A	0.982	−0.244	0.037	2.95 × 10^−10^	1.36 × 10^−8^	3
TR	*TPBG*	rs77834841	NC_000006.11:g.83135354C>T	0.976	−0.233	0.035	3.08 × 10^−10^	1.36 × 10^−8^	4

Trait: DP or TR phenotype found in association with the gene. Genes: closest gene to the top SNP obtained by VEP. Top SNP: most significant SNP; rsIDs are updated to dbSNP build 144; HGVS nomenclature: variant nomenclature according to Human Genome Variation Society recommendations; genomic data is aligned to the GRCh37/hg19 reference sequence. Freq: frequency of the effect allele. Beta: effect from the GWAS analysis. StdErr: standard error of the Beta. *p*-value from GWAS analysis. FDR-p: *p*-value after FDR correction. N° of surrounding SNPs (*p* < 1 × 10^−4^): number of SNPs with *p* < 1 × 10^−4^ found in linkage with the top SNP.

After FDR correction, three genes resulted significantly associated with the DP phenotype: *PIWIL4* (top SNP rs118136100, FDR-p = 9.01*10^–9^), *MBD2* (top SNP rs17292725, FDR-p = 4.98*10^–8^) and *NTN1* (top SNP rs117797822, FDR-p = 4.97*10^–8^). Moreover, 15 genes resulted significantly associated with TR, with *NMB* (top SNP rs145079583, FDR-p = 8.34*10^–11^), *IQGAP1* (top SNP rs117555778, FDR-p = 6.52*10^–10^), *NPTN* (top SNP rs192430987, FDR-p = 6.52*10^–10^) and *ATF7IP2* (top SNP rs146729070, FDR-p = 6.52*10^–10^) being the most significantly associated genes.

### 3.2 Expression pattern of the associated genes in the human eye

**FIGURE 1:**
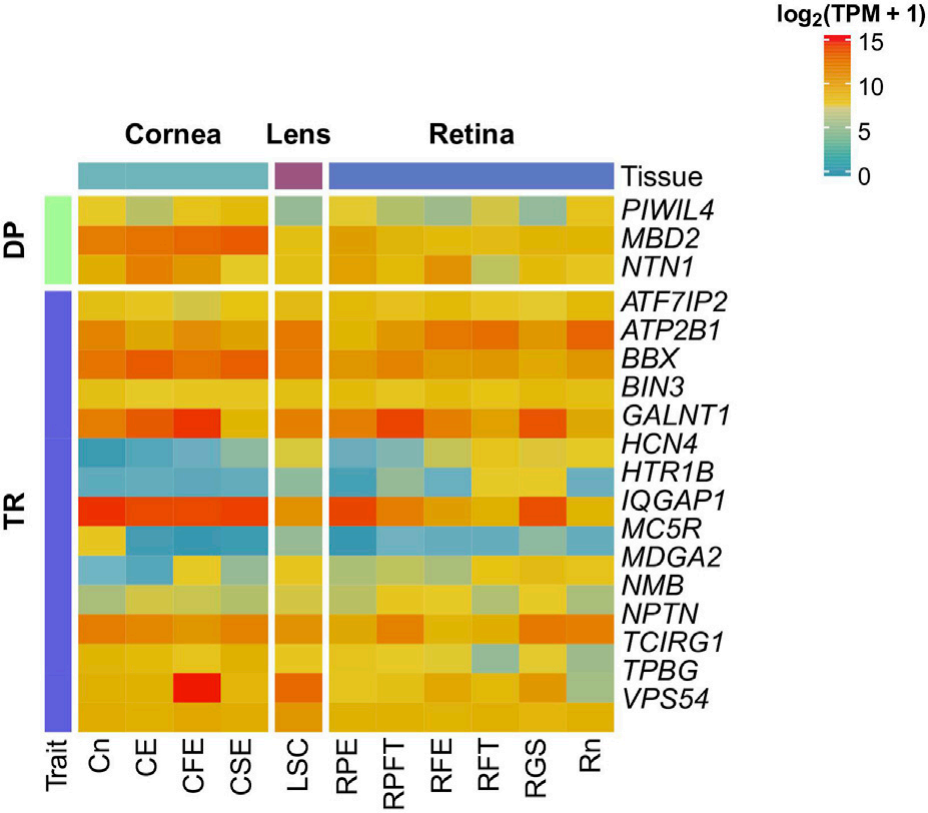
Gene expression of candidate genes in the eye. Gene expression of candidate genes was investigated in 11 healthy ocular tissues from Cornea, Lens, and Retina using the Eye on a Disk dataset. Candidate genes are at least weakly expressed in all the tissues. CE, Corneal Endothelium; CFE, Corneal Fetal Endothelium; CSE, Corneal Endothelial Stem Cells; Cn, Cornea; LSE, Lens Stem Cell Line; RFE, Fetal Eye Retina; RFT, Retinal Fetal Tissue; RGS, Retinal Ganglion Stem Cells; RPE, Retinal Pigment Epithelium; RPFT, Fetal RPE; Rt, Retina.

The expression patterns in the human eye of the three genes for DP and the 15 for TR were investigated using expression data from EiaD and are shown in Figure 1. The corresponding values of log_2_ (TPM+1) are fully reported in Supplementary Table S4.

All genes displayed at least a weak expression in all inquired tissues. As regards DP, *NTN1* and *MBD2* showed high expression (log_2_ (TPM+1) > 10) throughout all the tissues, especially in corneal tissues. Conversely, *PIWIL4* showed weak expression (log_2_ (TPM+1) < 5) in all tissues, excluding corneal tissues, RPE and Rn. Concerning TR, *VPS54*, *BIN3*, *TCIRG1*, *ATF7IP2*, *ATP2B1*, *BBX*, *GALNT1* and *IQGAP1* showed a high expression in all tissues. In contrast, *MC5R*, *MDGA2*, *HTR1B* and *HCN4* showed weak expression in almost all investigated tissues.

### 3.3 Pathway analysis

**FIGURE 2:**
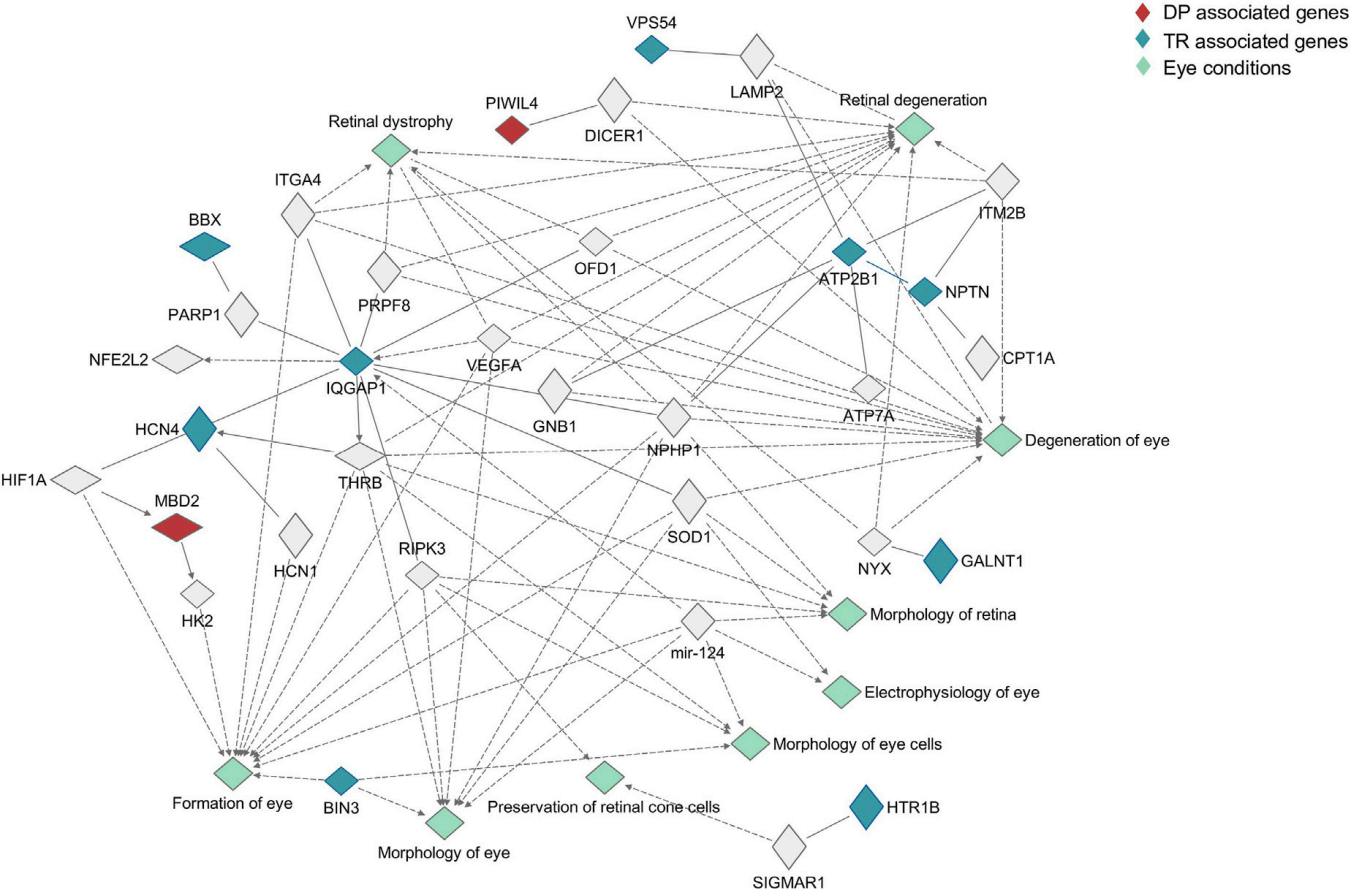
Pathway analysis of candidate genes. The role played in the eye by candidate genes was investigated using Ingenuity Pathway analysis, searching for interaction between candidate genes and a list of more than 500 genes involved in eye physiology and pathology. Genes associated with Deutan-Protan (DP) are highlighted in red, while genes associated with Tritan (TR) are highlighted in blue. Eye conditions associated with the interactions are highlighted in aquamarine.

Finally, the interaction between the resulting genes for both traits and a set of more than 500 genes involved in eye pathology and physiology was investigated using IPA’s Path Explorer. The interaction network is shown in Figure 2. The complete list of genes is reported in Supplementary Table S5.

With the exception of seven genes, *TPBG*, *TCIRG1*, *MDGA2*, *ATF7IP2*, *NTN1*, *MC5R*, and *NMB*, associations with genes involved in eye morphogenesis (formation of the eye, morphology of the eye and retina, morphology of eye cells), physiology (preservation of cone cells, electrophysiology of the eye) and degeneration (retinal dystrophy, retina degeneration, degeneration of the eye) have been found. Concerning DP, *MBD2* was involved in the formation of the eye, interacting both with *HK2* and *HIF1A*, while *PIWIL4* displayed an interaction with *DICER1*, which was involved in retinal degeneration. With regards to TR, *IQGAP1* and *ATP2B1* showed the most complex networks of interaction, interacting both with other candidate genes and with genes involved in all the investigated processes.

## 4 Discussion

In this study, we performed the first GWAS analyses investigating the molecular determinants of multifactorial forms of CVDs in the rare Silk Road cohort. Our analyses led to the identification of 18 candidate genes. Among them, seven genes proved to be particularly interesting, considering their expression data and pathway analysis results, as described below.

Concerning DP, three genes: *PIWIL4*, *MBD2*, and *NTN1*, were significantly associated after the FDR correction. The *PIWIL4* gene encodes for a protein belonging to the Argonaute family of proteins. Piwi proteins are believed to play a crucial role in spermatogenesis, as their absence leads to male infertility. This gene showed strong expression values in corneal tissues (Cn, CFE, CSE), in the retinal pigmented epithelium, and in the retina. *PIWIL4* is reported to regulate the expression of Alu RNA in response to oxidative stress, protecting RPE from degeneration (Hwang et al., 2019). Interestingly, the pathway analysis showed *PIWIL4* interaction with *DICER1*, and deficiency of this gene in response to oxidative stress in RPE is reported to lead to the accumulation of Alu RNA and degeneration of the RPE (Kaarniranta et al., 2020), suggesting a role of both *PIWIL4* and *DICER1* in the preservation of RPE. Moreover, *PIWIL4* modulates the expression of tight-junctions proteins in RPE, contributing to the structural integrity of the epithelium (Sivagurunathan et al., 2017).

MBD2 encodes for the methyl-CpG binding domain protein 2, a protein that binds specific methylated sequences, involved in transcription regulation, both repressing and activating the transcription of methylated sites. Moreover, MBD2 encoded protein is associated with many types of cancer, such as breast (Liu et al., 2021), renal (Li et al., 2020), colorectal (Yu et al., 2020) and prostate (Patra et al., 2003). Furthermore, MBD2 is reported to be involved in the T-cells development (Cheng et al., 2021). This gene showed a high expression in all investigated tissues, particularly in corneal tissues. MBD2 protein is reported to mediate apoptosis in retinal ganglion cells (Ge et al., 2020), and possibly play a role in the pathogenesis of age-related macular degeneration (Pan et al., 2014). In addition, our pathway analyses showed a link between MBD2 protein and hexokinase 2 (HK2) protein in the eye’s formation process. HK2 protein is reported to have several crucial roles in maintaining photoreceptors’ health and functionality (Weh et al., 2020).

NTN1 encodes for Netrin 1, a laminin-related secreted protein involved in axon guidance, cell migration, morphogenesis, angiogenesis, peripheral nerve regeneration and Schwann cell proliferation. NTN1 showed high expression values throughout all inquired tissues, especially in CE, CFE, RFE, and RPE. Although no association between NTN1 and genes involved in eye physiology and pathology was found by the pathway analysis, in the eye, netrin-1 is reported as a key molecule for the growth of axons directed to the optical nerve head (Mann et al., 2004) and for the migration of the glial-precursors from the brain to the retina (Tsai and Miller, 2002).

Notably, all the above genes could play a role in determining the DP phenotype. Alterations in PIWIL4 could lead to RPE degeneration, thus hindering its ability to support photoreceptors and correct color vision. Concerning MBD2 and NTN1, both of these genes play a role in the transmission of the visual signal, acting in retinal ganglion cells and in the optic nerve, respectively. We can therefore hypothesize that defects in these genes could alter the functionality of these tissues leading to an altered color vision.

With regards to TR, among those significantly associated after the FDR correction, four genes - VPS54, IQGAP1, NMB, and MC5R - stood out as promising candidates, considering the number of SNPs in linkage with the top SNP, their reported role in the eye, and their expression values. VPS54 encodes for a subunit of the GARP complex, a protein complex involved in retrograde transport from early and late endosomes to the trans-Golgi network (TGN). Moreover, VPS54 is reported to be part of RP28, a locus associated with recessive retinitis pigmentosa (Kumar et al., 2004). VPS54 showed a consistent expression in all investigated tissues, and the pathway analysis highlighted its interaction with LAMP2. Loss of function variants in LAMP2 cause Danon disease, a glycogen storage disease also known as X-linked vacuolar cardiomyopathy and myopathy. However, LAMP2 deficiencies are also reported to cause retinopathies and RPE degeneration independently (Kousal et al., 2021) or in association with Danon disease (Fukushima et al., 2020). Interestingly *VPS54* knock-out and the resulting GARP deficiency are reported to alter the formation of lysosomal structures deputed to the accumulation of endolysosomal proteins, including Lamp2 (D’Souza et al., 2019).

IQGAP1 belongs to the IQGAP protein family, a family of scaffold proteins involved in the regulation of several cellular pathways and functions. In particular, the isoform encoded by IQGAP1 interacts with phosphatidylinositol phosphate kinase mediating cell motility and Akt phosphorylation. IQGAP1 showed high expression values throughout all tissues, especially in corneal tissues, in RPE and RGS, and showed a complex network of interactions in the pathway analysis. For instance, IQGAP1 interacts with THRB, a gene encoding for the thyroid hormone receptor (TRβ), and is reported to enhance transcriptional activation of TRβ isoform 2 (Hahm and Privalsky, 2013). The THRB gene is involved in cone photoreceptors’ development and function (Deveau et al., 2020). Indeed, THRB is reported to inhibit S opsin expression (Ma and Ding, 2016), and its mutation leads to an imbalance in the distribution of cone types in mice retina (Ng et al., 2001). Moreover, IQGAP1 is reported to regulate retinal neurite growth in chicken retina (Oblander and Brady-Kalnay, 2010) and to play a key role in the regulation of choroidal endothelial cells in the pathogenesis of Age-related Macular Degeneration (Ramshekar et al., 2021).

NMB encodes for neuromedin B, a member of the bombesin-related family of neuropeptides. Neuromedin B is expressed in the central nervous system and in the gastrointestinal tract and is involved in several physiological functions, including regulation of exocrine and endocrine secretions, smooth muscle contraction, feeding, blood pressure, blood glucose, body temperature, and cell growth. NMB showed a moderate expression in all tissues, with higher levels in RPFT, RFE, and RGS. Although no associations between the inquired genes and this gene were highlighted by the pathway analysis, NMB is reported to play a role in the induction of intracellular Ca^2+^ transient and phosphatidylinositol turnover in RPE cells (Kuriyama et al., 1992) and to constitute a marker of chicken retinal ganglion cells (Yamagata et al., 2006).

MC5R belongs to the family of melanocortin receptors, formed by five G-protein coupled receptors involved in a broad spectrum of physiological processes, including regulation of pigmentation, regulation of inflammation, and pain perception. MC5R has a wide peripheral distribution and is involved in many processes such as glucose uptake (Enriori et al., 2016), exocrine gland secretion (Chen et al., 1997) and fatty acids oxidation (An et al., 2007). This gene showed poor expression in all inquired tissues, excluding the corneal tissue, and no associations were found for this gene through the pathway analysis. However, MC5R is reported to protect from uveitis-derived retinal damage by regulating the activation of CD4^+^ regulatory T cells (Taylor et al., 2006) and to attenuate retinal degeneration in an experimental model of diabetic retinopathy (Rossi et al., 2016). Moreover, MC5R is reported to play a crucial role in the lacrimal gland, maintaining lacrimal function and secretion (Nguyen et al., 2004).

All the above genes proved to be potentially involved in TR determination: defects in *VPS54* could lead to RPE degeneration, thus leading to color vision impairment; alterations in *IQGAP1* can cause cone type imbalances, with a diminished number and functionality of S cones; defects in NMB may alter RPE’s ability to regulate fluid homeostasis, resulting in subretinal fluid accumulation and retinal function impairment; alterations in *MC5R* could affect lacrimal gland function leading to dry eye onset and progressive visual impairment.

In conclusion, this study highlighted, for the first time, the association of 15 new genes with the determination of multifactorial forms of CVDs. Notably, seven genes, *PIWIL4*, *MBD2*, *NTN1*, *VPS54*, *IQGAP1*, *NMB*, and *MC5R*, proved to be particularly promising considering their expression in the eye and their known implication in several physiologic and pathologic eye processes. It is worth noting how, in our study, the availability of phenotypic and genotypic data from individuals coming from isolated communities allowed us to enhance the discovery power of GWAS, offering the possibility to capture new and impactful polymorphisms associated with complex traits. Up to date, there are few to no studies investigating complex traits in Silk Road populations. Therefore, with this work, we provide new and insightful information on such underrepresented populations and a natural next step of future research could seek to replicate these findings in different cohorts.

## Data Availability

Publicly available datasets were analyzed in this study. This data can be found here: European Variation Archive (EVA): https://www.ebi.ac.uk/eva/?eva-study=PRJEB60906.
