# Supplementary material for: Identifying missing pieces in color vision defects: a genome-wide association study in Silk Road populations
Source: Front Genet. 2023 Jun 9;14:1161696. doi: 10.3389/fgene.2023.1161696 (PMC10288324; doi:10.3389/fgene.2023.1161696)

Supplementary Figure 2: Manhattan and QQplots for both DP and TR traits. A) Manhattan plot for DP trait; B) QQplot for DP trait; C) Manhattan plot for TR trait; D) QQplot for TR trait. All the p-values represented in the figures are raw p-values resulting from the analyses, without any correction.

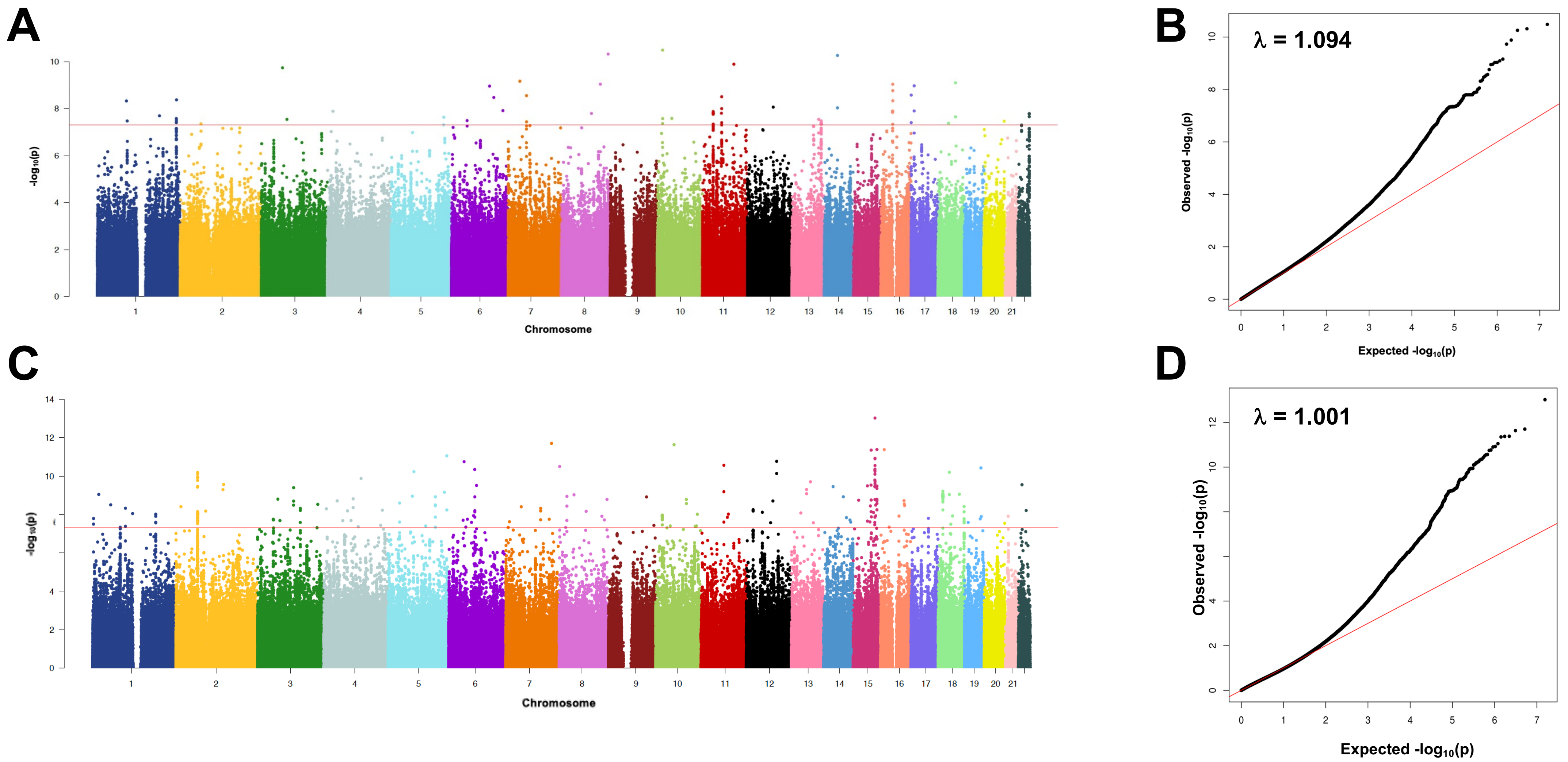

Supplement: Supplementary file 3 [file Image2.PDF]
