# Supplementary material for: Identifying missing pieces in color vision defects: a genome-wide association study in Silk Road populations
Source: Front Genet. 2023 Jun 9;14:1161696. doi: 10.3389/fgene.2023.1161696 (PMC10288324; doi:10.3389/fgene.2023.1161696)

Supplementary Figure 1: Farnsworth D-15 diagram. Numbers represent the colored disks. Lines in the center define the type and intensity of CVD: the type is determined based on the first line parallel to one of the colored axes to cross the center. The number of lines parallel to one of the axis intersecting the center defines CVDs severity. A) Normal vision diagram; B) Severe deutanomaly diagram; C) Severe protanomaly diagram; D) Severe tritanomaly diagram.

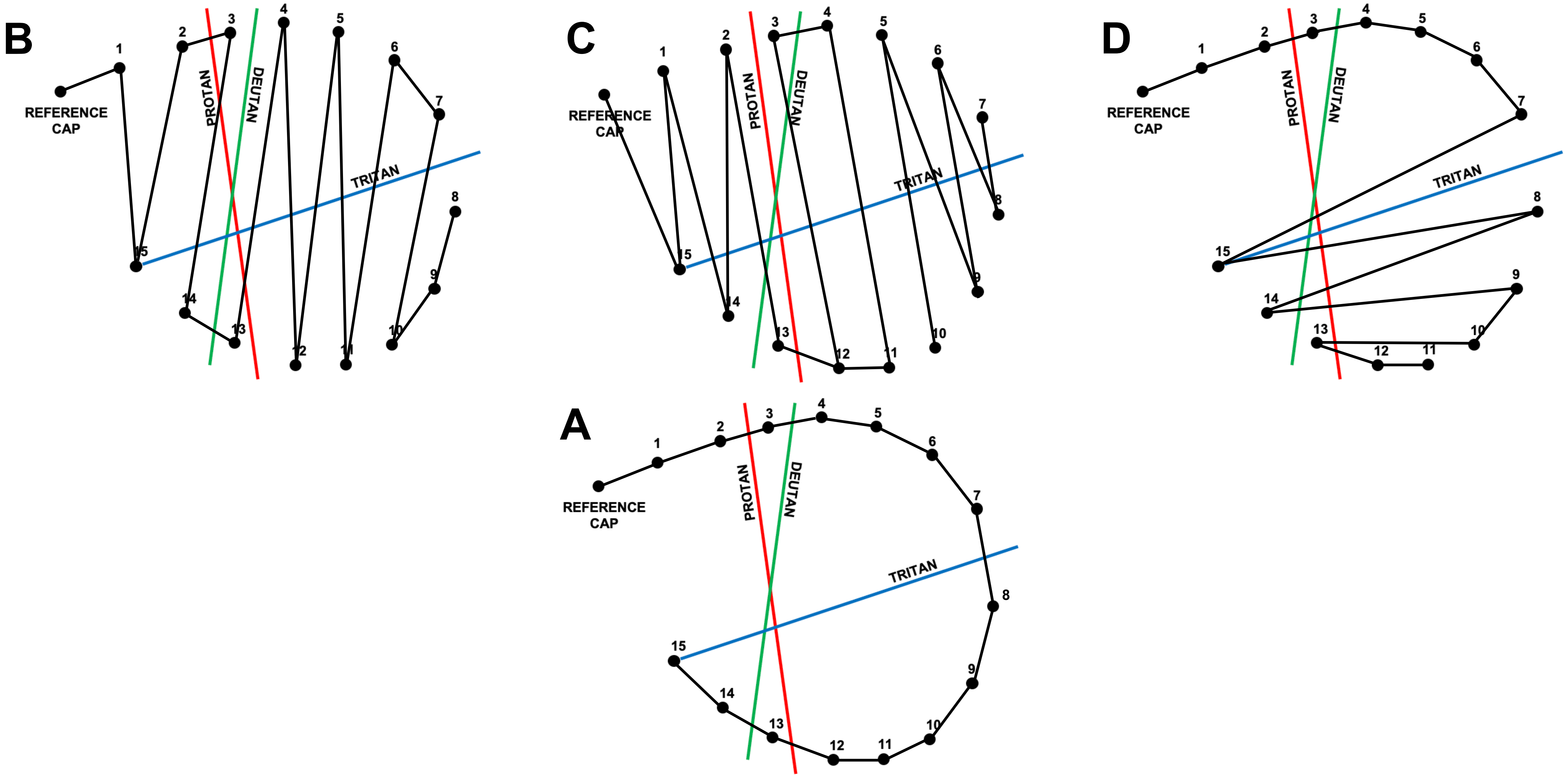

Supplement: Supplementary file 7 [file Image1.PDF]
